# Supplementary material for: Comparison of induced neurons reveals slower structural and functional maturation in humans than in apes
Source: eLife. 2021 Jan 20;10:e59323. doi: 10.7554/eLife.59323 (PMC7870144; doi:10.7554/eLife.59323)
Supplement: Supplementary file 1. [file elife-59323-supp1.docx]

|  | d7 | | | | d14 | | | | d21 | | | | d35 | | | |
| --- | --- | --- | --- | --- | --- | --- | --- | --- | --- | --- | --- | --- | --- | --- | --- | --- |
| cellline/ species | uni | bi | multi | total | uni | bi | multi | total | uni | bi | multi | total | uni | bi | multi | total |
| hiPS-409-B2 | 18 | 34 | 24 | 76 | 2 | 10 | 21 | 33 | 1 | 9 | 29 | 39 | 1 | 4 | 11 | 16 |
| SC102A1 | 1 | 37 | 29 | 67 | 0 | 8 | 15 | 23 | 1 | 8 | 20 | 29 | 0 | 0 | 14 | 14 |
| H9 | 2 | 21 | 31 | 54 | 2 | 15 | 13 | 30 | 1 | 12 | 44 | 57 | 0 | 0 | 3 | 3 |
| Human | 21 | 92 | 84 | 197 | 4 | 33 | 49 | 86 | 3 | 29 | 93 | 125 | 1 | 4 | 28 | 33 |

|  | d7 | | | | d14 | | | | d21 | | | | d35 | | | |
| --- | --- | --- | --- | --- | --- | --- | --- | --- | --- | --- | --- | --- | --- | --- | --- | --- |
| cellline/ species | uni | bi | multi | total | uni | bi | multi | total | uni | bi | multi | total | uni | bi | multi | total |
| SandraA | 4 | 30 | 26 | 60 | 0 | 9 | 19 | 28 | 1 | 9 | 27 | 38 | 1 | 5 | 16 | 22 |
| JoC | 2 | 6 | 6 | 14 | 0 | 7 | 31 | 38 | 1 | 6 | 24 | 31 | 0 | 1 | 10 | 11 |
| BmRNA | 3 | 2 | 13 | 18 | 1 | 5 | 28 | 34 | 2 | 7 | 30 | 39 | 0 | 3 | 6 | 9 |
| ChimpanzeeBonobo | 9 | 38 | 45 | 92 | 1 | 21 | 78 | 100 | 4 | 22 | 81 | 108 | 1 | 9 | 32 | 42 |
