# Supplementary material for: Comparison of induced neurons reveals slower structural and functional maturation in humans than in apes
Source: eLife. 2021 Jan 20;10:e59323. doi: 10.7554/eLife.59323 (PMC7870144; doi:10.7554/eLife.59323)
Supplement: Supplementary file 2. [file elife-59323-supp2.docx]

**Supplementary Table 2**

Schoernig at al.

| REAGENT or RESOURCE | | SOURCE | | IDENTIFIER | |
| --- | --- | --- | --- | --- | --- |
|  | |  | | |  |
| CONSUMABLES | |  | |  | |
| Cell culture test plates, sterile  (6 well, 24 well) | | TPP® | | 92006, 92024 | |
| Coverslips (pre-treated, 12 mm) | | Kleinfeld Labortechnik | | GG-12-Pre | |
| Cryogenic vials | | Thermo Fisher Scientific | | 5000-0020 | |
| Falcon tubes (15 ml, 50 ml) | | Greiner bio-one | | 188271, 210261 | |
| Glass Pasteur pipettes | | VWR | | 612-1701 | |
| Lens cleaning tissue | | GE Healthcare Life Sciences | | 2105-841 | |
| Microscope slides | | Thermo Fisher Scientific | | J3800AMNZ | |
| Parafilm | | Carl Roth | | PM-996 | |
| Pipette tips, filter tips | | Eppendorf, Gilson | |  | |
| Precision wipes | | Kimtech science | | 7552 | |
| Serological pipets, sterile  (5 ml, 10 ml, 25 ml, 50 ml) | | Corning® | |  | |
| Single-use syringes  (5 ml, 10 ml, 30 ml) | | B Braun | |  | |
| Sterile square media bottle  (125 ml, 250 ml) | | Nalgene | | 2019-0125, 2019-0250 | |
| Syringe-Filter (0.22 µm) | | TPP® | | 99722 | |
| Tissue culture dish (40 mm) | | TPP® | | 93040 | |
| Tissue culture flask, sterile (75 cm²) | | TPP® | | 90076 | |
| Tubes (0.5 ml, 1.5 ml, 2.0 ml) | | Eppendorf | |  | |
|  | |  | |  | |
| DEVICES | |  | |  | |
| Accu-jet® pro | | Brand | |  | |
| Analytical balance | | Kern | | AEJ-CM | |
| Beaker (100 ml, 500 ml) | | Schott Duran | |  | |
| Bioanalyzer Instrument 2100 | | Agilent | | G2939Ba | |
| Centrifuge | | Thermo Fisher Scientific | | Heraeus Megafuge 40R | |
| Clean bench | | Thermo Fisher Scientific | | Heraeus Instruments | |
| Confocal laser scanning microscope | | OLYMPUS  FV1200  Fluorescent Light Source: Power Supply: | | BX61W1 Multiphoton  FV1000 Microscope  U-HGLGPS  FV10-MCPSU | |
| Confocal laser scanning microscope | | Zeiss LSM 780 NLO | | Zeiss axio Examiner.Z1, upright stand | |
| Countess™ automated cell counter | | Invitrogen | |  | |
| EPC-10 amplifier | | HEKA, Lambrecht, Germany | |  | |
| Freezing container | | Nalgene | |  | |
| Glass bottles (100 ml, 500 ml) | | Schott Duran | |  | |
| Glass electrodes for electrophysiology | | Hilgenberg, Germany | | 4.5-5.5 MΩ, oD/iD, borosilicate | |
| HighSeq2500 | | Ilumina | |  | |
| Incubator Heracell 240 | | Thermo Fisher Scientific | |  | |
| Inverse microscope | | Zeiss MultiSpec-Micomager DualV  Filter Set: | | Axiovert 200  46HEYFP | |
| Inverted microscope | | Zeiss  Fluorescent Light Source: | | Axio Observer.Z1 SMC200  HXP12OV  232 | |
| Magnetic stirring hot plate | | Heidolph | | MR3002 | |
| Microcentrifuge | | Carl Roth | |  | |
| Nucleofector™2b | | Lonza | |  | |
| Pipettes (10 µl, 100 µl, 200 µl, 1000 µl) | | Eppendorf, Gilson | |  | |
| Spinning disc confocal microscope | | Andor Revolution WD Borealis Mosaic | | Andor IX 83, inverted stand | |
| Stereo microscope | | Olympus | | SZX7 | |
| Tweezers, Scoops | |  | |  | |
| Upright microscope for electrophysiology | | Olympus | | BXW-51 | |
| Vacusafe™ Vacuum aspiration system | | Integra Biosciences | |  | |
| Vortex-Genie 2 | | Scientific Industries | |  | |
|  | |  | |  | |
| REAGENTS | |  | |  | |
| Accutase® solution | | Sigma Aldrich | | A6964 | |
| B27 Supplement (50x) | | Gibco® | | 17504-044 | |
| BDNF, human | | Promokine | | C-66212 | |
| Borax | | Sigma Aldrich | | B3545 | |
| Boric acid | | Merck | | 1.00165.1000 | |
| CaCl_2_ | | Merck | |  | |
| Chromium Single Cell 3’ Library & Gel Bead Kit v2, 16 rxns | | 10X Genomics | | PN-120237 | |
| Cytosine β-D-arabinofuranoside | | Sigma Aldrich | | C1768 | |
| DAPI | | Sigma Aldrich | | D9542 | |
| DMEM, high glucose | | Gibco® | | 11965-092 | |
| DMEM/F-12 | | Gibco® | | 31330-038 | |
| Dnase I | | New England Biolabs | | 2 U/µl | |
| Doxycycline | | Sigma Aldrich | | D9891 | |
| Dulbecco’s Phosphate-Buffered Saline (DPBS) | | Gibco® | | 14190-094 | |
| Ethylenediaminetetraacetic acid (EDTA) | | Gibco® | | 15575-038 | |
| Ethanol | | Carl Roth | | K928.1 | |
| Fetal bovine serum | | Sigma Aldrich | | F2442 | |
| G418 disulfate salt solution | | Sigma Aldrich | | 68168 | |
| Gelatine | | Carl Roth | | 4308.1 | |
| Glucose | | Sigma-Aldrich | | 158968-500G | |
| GlutaMAX | | Gibco® | | 35050-038 | |
| Glycine | | Serva | | 23390.03 | |
| HEPES | | Sigma-Aldrich | | H3375-250G | |
| Hygromycin B-solution | | Carl Roth | | CP12.2 | |
| Isopropanol | | Merck | | 1.09634.2511 | |
| KCl | | Merck | |  | |
| K-gluconate | | Merck | |  | |
| Knockout DMEM/F12 | | Gibco® | | 12660-012 | |
| Kynurenic acid | | Sigma Aldrich | | K3375 | |
| Laminin | | Sigma Aldrich | | L2020 | |
| Lipofectamine 3000 Transfection Kit | | Invitrogen | | C3000-008 | |
| Matrigel Matrix | | Corning® | | 354277 | |
| MEM non-essential amino acid solution (100x) | | Sigma Aldrich | | M7145 | |
| mFreSR™ | | Stemcell™ Technologies | | 05853 | |
| MgCl_2_ | | Merck | |  | |
| Mowiol 4-88 | | Sigma Aldrich | | 81381 | |
| mTeSR™1 | | Stemcell™ Technologies | | 85851 | |
| mTeSR™1 5x Supplement | | Stemcell™ Technologies | | 85852 | |
| N2-Supplement (100x) | | Thermo Fisher Scientific | | 17502048 | |
| NaCl | | Merck | | 1.06404.1000 | |
| NaHCO_3_ | | Sigma-Aldrich | | S5761-1KG | |
| Na_2_HPO_4_ • 2 H_2_O | | Merck | | 1.06580.1000 | |
| NaH_2_PO_4_ • H_2_O | | Merck | | 1.06346.1000 | |
| NaOH | | Merck | | 1.06462.1000 | |
| Neurobasal™ Medium | | Gibco® | | 21103-049 | |
| NT3, human | | Promokine | | C-66425 | |
| Opti-MEM™ | | Gibco® | | 31985-070 | |
| Paraformaldehyde, 96 % | | ACROS Organics™ | | 30525-89-4 | |
| Pen/Strep | | Gibco® | | 15140-122 | |
| Percoll | | Sigma-Aldrich | | P1644-100ML | |
| Phosphate-Buffered Saline (PBS) | | Gibco® | | 10010-015 | |
| Poly-D-Lysine solution | | Sigma Aldrich | | A-003-E | |
| Puromycin dihydrochloride | | Sigma Aldrich | | P9620 | |
| Rock-Inhibitor Y-27632 | | Stemcell™ Technologies | | 72305 | |
| Sucrose | | Merck | | 1.07653.100 | |
| Triton X-100 Solution | | Sigma Aldrich | | 93443 | |
| TrypLE™ Express (1x) | | Gibco® | | 12605-010 | |
| UltraPure™ Destilled Water | | Invitrogen | | 10977-035 | |
|  | |  | |  | |
| ANTIBODIES | |  | |  | |
| Anti MAP2 Antibody | | Invitrogen | | PA1-16751  Dilution 1:1000 | |
| Anti-Synapsin 1-2 guineapig antiserum | | Synaptic Systems | | 106004  Dilution 1:1000 | |
| Purified anti-Tubulinβ3 (TUBB3), Clone: TUJ1  Coupled to AlexaFlour^TM^ 488 or Alexa Flour^TM^ 555 | | BioLegend | | 801202  Dilution 1:1000 | |
| Purified anit-Neurofilament Marker (pan-axonal, cocktail), Clone: SMI312 | | BioLegend | | 8379074  Dilution 1:400 | |
| Anti-ISL1 Monoclonal Antibody (1H9) | | Invitrogen | | MA5-15515  Dilution 1:1000 | |
| Anti-Peripherin antibody | | Abcam | | ab39374  Dilution 1:1000 | |
| Anti-BRN2 antibody (goat) | | Santa Cruz | | sc-6029  Dilution 1:100 | |
| Anti-TBR1 antibody (rabbit) | | Abcam | | Ab31940  Dilution 1:200 | |
| Anti-CUX1 antibody (mouse) | | Santa Cruz | | sc-514008  Dilution  1:500 | |
| Donkey anti-Mouse IgG (H+L) Highly Cross-Adsorbed Secondary Antibody, Alexa Fluor^TM^ 555 | Invitrogen | | | A21202  Dilution 1:1000 | |
| Goat anti-Rabbit IgG (H+L) Highly Cross-Adsorbed Secondary Antibody, Alexa Fluor^TM^ Plus 555 | Invitrogen | | | A32732  Dilution 1:1000 | |
| Goat anti-Chicken IgG Highly Cross-Adsorbed Secondary Antibody, Alexa Fluor^TM^ 488 | Invitrogen | | | A11039  Dilution 1:1000 | |
| Goat anti-Guinea Pig IgG Highly Cross-Adsorbed Secondary Antibody, Alexa FluorTM 568 | Invitrogen | | | A11075  Dilution 1:1000 | |
|  | |  | |  | |
| CELLS | |  | |  | |
| Human | | 409B2 iPS cell line  409B2_Ngn2 | | Riken BRC Cellbank  Generated by Maria Schörnig | |
| Human | | SC102A-1 iPS cell line  SC102A-1_Ngn2 | | Systems Biosciences  Generated by Maria Schörnig | |
| Human | | HmRNA iPS cell line  HmRNA_Ngn2 | | Generated by Anne Weigert  Generated by Anne Weigert | |
| Human | | H9 ES cell line  H9_Ngn2 | | WiCell  Generated by Maria Schörnig | |
| Chimpanzee | | Sandra A  Sandra A_Ngn2 | | Generated in a previous study.  Generated by Maria Schörnig | |
| Chimpanzee | | Jo_C  Jo_C_Ngn2 | | Generated in a previous study.  Generated by Maria Schörnig | |
| Chimpanzee | | ciPS01  Chimp male iPSC Sendai CL5  ciPS01_Ngn2 | | Provided by the Max-Delbrück-Centrum für Molekulare Medizin  Generated by Anne Weigert. | |
| Bonobo | | BmRNA  BmRNA_Ngn2 | | Reprogramming by mRNA  Generated by Maria Schörnig | |
| Primary Rat Astrocytes | | RjHan:WI – Wistar rat from Janvier | | Primary cortical rat astrocytes were freshly prepared for this study | |
| Human dermal fibroblasts | | Lonza | | CC-2511 | |
|  | |  | |  | |
| PLASMIDS | |  | |  | |
| pmax GFP | | Lonza | | D-00072 | |
| pLVX-EF1α-(Tet-On-Advanced)-IRES-G418(R) | | Provided by Nael Nadif Kasri | |  | |
| pLVX-(TRE-thight)-(MOUSE)Ngn2-PGK-Puromycin(R) | | Provided by Nael Nadif Kasri | |  | |
|  | |  | |  | |
| SOFTWARE | |  | |  | |
| AxioVision Rel. | | 4.8 | | | |
| FV10-ASW | | 4.2 | | | |
| Image J | | v1.51w. | | | |
| Imaris | | 9.5. | | | |
| Imaris File Converter | | 9.2.0 | | | |
| Imaris Stitcher | | 9.2.0 | | | |
| Origin | | OriginLab version 2018-2019b | | | |
| Patch-and Fitmaster software | | HEKA version 2.9x | | | |
| R | | 3.5.1 | | | |
| ZEN | |  | | | |
|  | |  | | | |
| SOLUTIONS, BUFFERS AND MEDIA | |  | | | |
| PFA 4 % | | 4% PFA  4% Sucrose  120 mM sodium phosphate buffer pH 7.4 | | | |
|  | |  | | | |
| Sodium phosphate buffer  (240mM) | | 240 mM Na_2_HPO_4_  240 mM NaH_2_PO_4_, pH 7.4 | | | |
|  | |  | | | |
| Astrocyte medium | | DMEM high glucose  10% FBS  1% Pen/Strep | | | |
|  | |  | | | |
| Borate-Buffer, pH 8.4 | | boric acid  borax | | | |
|  | |  | | | |
| Immunofluorescence buffer  (IF buffer) | | 30 mM NaCL  0.2% gelatine  0.05% Triton X-100  120 mM phosphate buffer | | | |
|  | |  | | | |
| Glycine buffer | | 0.2 M glycine  120 mM phosphate buffer | | | |
|  | |  | | | |
| Permeabilization buffer | | 0.05% Triton X-100  120 mM phosphate buffer | | | |
|  | |  | | | |
| Artificial cerebrospinal fluid | | 100 mM NaCl  3.5 mM KCl  1 mM MgCl_2_  2 mM CaCl_2_  30 mM NaHCO_3_  1.25 mM NaH_2_PO_4_  10 mM glucose | | | |
|  | |  | | | |
| Internal solution electrophysiology | | 130 mM K-gluconate  10 mM NaCl  4 mM Mg-ATP  0.5 GTP  10 mM HEPES  0.05 mM EGTA | | | |
|  | |  | | | |
| KITs | |  | | | |
| Stem MACS mRNA transfection Kit | | Miltenyi Biotec | 130-104-463 | | |
| Human Pluripotent Stem Cell 3 Colour Immunohistochemistry Kit | | R&D Systems1 | SC021 | | |
| Human Pluripotent Stem Cell Functional Identification Kit | | R&D Systems | SC027B | | |
| StemMACS Trilineage Differentiation Kit | | Miltenyi Biotec | 130-115-660 | | |
