# Supplementary material for: Comparison of induced neurons reveals slower structural and functional maturation in humans than in apes
Source: eLife. 2021 Jan 20;10:e59323. doi: 10.7554/eLife.59323 (PMC7870144; doi:10.7554/eLife.59323)
Supplement: Supplementary file 3. [file elife-59323-supp3.docx]

| day | species | Vrpm | | | | N | p-val. | Rcell | Rcell | |  | Rcell | N | p | Ccell | Ccell | Ccell |  | N | p |
| --- | --- | --- | --- | --- | --- | --- | --- | --- | --- | --- | --- | --- | --- | --- | --- | --- | --- | --- | --- | --- |
|  |  | mV | mV | mV | mV | # | days | MΩ | MΩ | | MΩ | MΩ | # | days | pF | pF | pF |  | # | days |
|  |  | median | mean | SD | SEM |  | spec*days | median | mean | | SD | SEM |  | spec*days | median | mean | SD | SEM |  | spec*days |
| D2-4 | ape | -57 | -58 | 7,8 | 2,8 | 8 | F_days_ 0,992; p = 0,438 | 728 | 859 | | 569 | 201 | 8 | **F_days_ 12,1; p = 1,83 E-13** | 15,6 | 15,6 | 3,4 | 1,2 | 8 | **F_days_ 19,1; p = 7,45 E-21** |
|  | human | -73 | -67 | 17,0 | 4,9 | 12 |  | 657 | 744 | | 519 | 150 | 12 |  | 17,7 | 18,4 | 5,7 | 1,6 | 12 |  |
| D6-8 | ape | -66 | -63 | 14,0 | 3,5 | 16 |  | 1110 | 1100 | | 519 | 139 | 16 |  | 14,7 | 14,4 | 3,4 | 0,8 | 16 |  |
|  | human | -54 | -56 | 14,2 | 3,7 | 15 |  | 921 | 1140 | | 562 | 145 | 15 |  | 13,9 | 15,4 | 5,8 | 1,5 | 15 |  |
| D9-10 | ape | -51 | -56 | 16,6 | 5,9 | 8 |  | 781 | 953 | | 362 | 128 | 8 |  | 15,8 | 15,4 | 2,4 | 0,8 | 8 |  |
|  | human | -64 | -65 | 10,9 | 3,4 | 10 |  | 867 | 893 | | 241 | 80 | 9 |  | 15,8 | 15,9 | 4,2 | 1,3 | 10 |  |
| D14-16 | ape | -61 | -57 | 13,1 | 4,1 | 10 |  | 1068 | 1200 | | 486 | 154 | 10 |  | 21,0 | 22,3 | 6,3 | 2,0 | 10 |  |
|  | human | -60 | -56 | 13,6 | 3,8 | 13 |  | 793 | 789 | | 381 | 110 | 12 |  | 16,1 | 17,3 | 3,6 | 1,0 | 12 |  |
| D20-24 | ape | -70 | -66 | 11,5 | 1,9 | 37 |  | 719 | 772 | | 368 | 59 | 39 |  | 24,5 | 25,8 | 8,5 | 1,4 | 39 |  |
|  | human | -58 | -58 | 11,7 | 2,0 | 36 |  | 640 | 699 | | 258 | 43 | 36 |  | 21,2 | 21,4 | 5,4 | 0,9 | 36 |  |
| D27-31 | ape | -66 | -61 | 14,7 | 3,1 | 22 |  | 568 | 704 | | 363 | 73 | 25 |  | 29,7 | 32,6 | 13,3 | 2,7 | 25 |  |
|  | human | -59 | -55 | 10,7 | 2,2 | 23 |  | 575 | 646 | | 301 | 58 | 27 |  | 21,6 | 23,6 | 10,6 | 2,0 | 27 |  |
| D34-36 | ape | -63 | -59 | 12,7 | 2,7 | 23 |  | 462 | 523 | | 254 | 53 | 23 |  | 30,1 | 30,9 | 12,1 | 2,5 | 23 |  |
|  | human | -57 | -58 | 12,0 | 2,5 | 23 |  | 578 | 554 | | 207 | 43 | 23 |  | 29,9 | 29,5 | 8,6 | 1,8 | 23 |  |
| D>49 | ape | -53 | -56 | 11,4 | 3,0 | 15 |  | 378 | 420 | | 180 | 47 | 15 |  | 36,1 | 36,5 | 14,1 | 3,6 | 15 |  |
|  | human | -60 | -58 | 12,2 | 2,7 | 20 |  | 477 | 559 | | 270 | 60 | 20 |  | 28,0 | 30,3 | 11,4 | 2,6 | 20 |  |
| p val. (species / spec.*days) | | F_species_ 0,075; p = 0,784 | | | | | F_spec*days_ 2,04;  p = 0,0508 |  | | F_species_ 1,79; p = 0,183 | | | | F_spec*days_ 1,34; p = 0,233 |  | **F_species_ 5,39; p = 0,0209** | | | | F_spec*days_ 1,66; p = 0,117 |

| day | species | Tau | | | | N | p | APs | APs | APs | N | p | p | EPSCs | EPSCs | EPSCs | N | p | p |
| --- | --- | --- | --- | --- | --- | --- | --- | --- | --- | --- | --- | --- | --- | --- | --- | --- | --- | --- | --- |
|  |  | ms | ms | ms | ms | # | days | # | # | # | # |  | days | Hz | Hz | Hz | # |  | days |
|  |  | median | mean | SD | SEM |  | spec*days | median | mean | SD |  |  | spec*days | median | mean | SEM |  |  | spec*days |
| D2-4 | ape | 8,97 | 13,4 | 9,53 | 3,37 | 8 | F_days_ 151; p = 0,165 | 1 | 0,7 | 0,5 | 8 | 0,862 | **F_days_ 22,8; p = 1,98E-24** | no EPSCs measured | | | | | |
|  | human | 9,37 | 12,6 | 8,03 | 2,32 | 12 |  | 1 | 0,6 | 0,5 | 12 |  |  |  |  |  |  |  |  |
| D6-8 | ape | 14,3 | 16,0 | 10,4 | 2,61 | 16 |  | 1,5 | 1,8 | 1,5 | 16 | 0,359 |  |  |  |  |  |  |  |
|  | human | 13,6 | 18,3 | 13,8 | 3,56 | 15 |  | 1 | 1,4 | 0,9 | 15 |  |  |  |  |  |  |  |  |
| D9-10 | ape | 13,6 | 15,0 | 6,86 | 2,43 | 8 |  | 1,5 | 3,75 | 4,3 | 8 | 0,127 |  |  |  |  |  |  |  |
|  | human | 12,8 | 14,9 | 6,79 | 2,26 | 9 |  | 1 | 1,1 | 0,6 | 10 |  |  |  |  |  |  |  |  |
| D14-16 | ape | 23,7 | 26,9 | 12,9 | 4,09 | 10 |  | 9,5 | 9,9 | 6,1 | 10 | **0,021** |  | 0,30 | 0,27 | 0,025 | 11 | **0,045** | **F_days_ 12; p = 2,8E-09** |
|  | human | 12,3 | 14,0 | 9,27 | 2,67 | 12 |  | 1 | 4 | 4,6 | 13 |  |  | 0,05 | 0,08 | 0,024 | 16 |  |  |
| D20-24 | ape | 18,1 | 19,1 | 9,98 | 1,62 | 38 |  | 12 | 10,9 | 5,6 | 39 | **0,0002** |  | 2,91 | 1,39 | 0,024 | 52 | **1,5E-07** |  |
|  | human | 14,4 | 15,0 | 6,70 | 1,12 | 36 |  | 5 | 6,2 | 4,9 | 36 |  |  | 0,12 | 0,14 | 0,021 | 51 |  |  |
| D27-31 | ape | 17,6 | 18,8 | 6,42 | 1,31 | 24 |  | 12 | 11,9 | 5,4 | 25 | **0,0011** |  | 4,73 | 6,96 | 0,021 | 25 | **3,8E-07** |  |
|  | human | 12,1 | 14,8 | 8,33 | 1,60 | 27 |  | 7 | 7 | 4,9 | 27 |  |  | 0,28 | 0,34 | 0,026 | 30 |  |  |
| D34-36 | ape | 11,8 | 14,6 | 6,58 | 1,37 | 23 |  | 13 | 12,3 | 6,1 | 23 | **0,0085** |  | 12,42 | 5,55 | 0,027 | 31 | **0,019** |  |
|  | human | 14,0 | 16,3 | 8,20 | 1,71 | 23 |  | 7 | 8 | 4,4 | 23 |  |  | 0,52 | 1,10 | 0,026 | 30 |  |  |
| D>49 | ape | 12,8 | 13,8 | 4,86 | 1,25 | 15 |  | 15 | 13,3 | 6,6 | 15 | 0,54 |  | 9,38 | 3,03 | 0,028 | 20 | 0,22 |  |
|  | human | 12,7 | 15,9 | 8,66 | 1,94 | 20 |  | 13,5 | 11,8 | 7,4 | 20 |  |  | 2,13 | 1,25 | 0,027 | 26 |  |  |
| p val. (species / spec.*days) | | F_species_ 3,08; p = 0,0803 | | | | | **F_spec*days_ 2,36; p = 0,0234** | **F_species_ 22,7; p = 2,96E-6** | | | |  | F_spec*days_ 1,55; p = 0,150 | **F_species_ 41; p = 7,3E-10** | | | |  | F_spec*days_ 2,0; p = 0,093 |
