# Supplementary material for: Comparison of induced neurons reveals slower structural and functional maturation in humans than in apes
Source: eLife. 2021 Jan 20;10:e59323. doi: 10.7554/eLife.59323 (PMC7870144; doi:10.7554/eLife.59323)
Supplement: Supplementary file 4. [file elife-59323-supp4.docx]

**Statistics total neurite length of bipolar iNs.**

| Days of differentiation | Species | N | Differences  in % | Mean [µm] | SD  [µm] | P value  (species) |
| --- | --- | --- | --- | --- | --- | --- |
| d7 | ape | 38 | -13% | 1916 | 791 | **0.0278**  ***** |
|  | human | 92 |  | 1673 | 1056 |  |
| d14 | ape | 21 | -82% | 11184 | 17897 | **0.0003 ***** |
|  | human | 33 |  | 1960 | 1580 |  |
| d21 | ape | 22 | -8% | 16699 | 16947 | 0.4508 |
|  | human | 29 |  | 15415 | 14553 |  |
| d35 | ape | 9 | +1% | 46158 | 49394 | 0.4693 |
|  | human | 4 |  | 46839 | 38651 |  |
|  |  |  |  |  |  |  |
| Spariro test | **W** | **P value** |  |  |  |  |
|  | 0.89869 | 7.215e-12 |  |  |  |  |
|  |  |  |  |  |  |  |
| 2 Way Anova | **Df** | **Sum Sq** | **Mean Sq** | **F value** | **P value** |  |
| species | 1 | 5.447 | 5.4466 | 35.6988 | **8.262e-09** | *** |
| day | 3 | 31.325 | 10.4416 | 68.4379 | **<2.2e-16** | *** |
| Species*day | 3 | 2.070 | 0.6900 | 4.522 | **0.004176** | ** |
| Residual | 240 | 1661.75 | 6.924 |  |  |  |

**Statistics total neurite length of multipolar iNs.**

| Days of differentiation | Species | N | Differences in % | Mean [µm] | SD [µm] | P value  (species) |
| --- | --- | --- | --- | --- | --- | --- |
| d7 | ape | 45 | -19% | 2353 | 1160 | **0.0098**  ****** |
|  | human | 84 |  | 1905 | 1137 |  |
| d14 | ape | 78 | -49% | 8082 | 13560 | **0.0215**  ***** |
|  | human | 49 |  | 4093 | 5848 |  |
| d21 | ape | 93 | +10% | 22594 | 23493 | 0.1434 |
|  | human | 81 |  | 24884 | 22216 |  |
| d35 | ape | 40 | -6% | 35615 | 45846 | 0.4088 |
|  | human | 28 |  | 33325 | 40643 |  |
|  |  |  |  |  |  |  |
| Spariro test | **W** | **p-value** |  |  |  |  |
|  | 0.95237 | 1.384e-11 |  |  |  |  |
|  |  |  |  |  |  |  |
| 2 Way Anova | **Df** | **Sum Sq** | **Mean Sq** | **F value** | **P value** |  |
| species | 1 | 0.696 | 0.6958 | 3.1951 | 0.0749 |  |
| day | 3 | 77.186 | 25.7287 | 118.1547 | **<2e-16** | ******* |
| Species*day | 3 | 1.561 | 0.5202 | 2.3889 | 0.0681 |  |
| Residual | 490 | 106.700 | 0.278 |  |  |  |

**Statistics relative longest neurite (axon) length of multipolar iNs.**

| Days of differentiation | Species | N | Differences in % | Mean  [µm] | SD  [µm] | P value  (species) |
| --- | --- | --- | --- | --- | --- | --- |
| d7 | ape | 45 | -13% | 1242 | 543 | 0.3327 |
|  | human | 84 |  | 1073 | 541 |  |
| d14 | ape | 78 | -28% | 3436 | 4621 | **0.0294**  ***** |
|  | human | 49 |  | 2461 | 4033 |  |
| d21 | ape | 93 | +20% | 10008 | 9040 | 0.0587 |
|  | human | 81 |  | 12045 | 9948 |  |
| d35 | ape | 40 | +25% | 12271 | 11318 | **0.0094**  ****** |
|  | human | 28 |  | 15330 | 12689 |  |
|  |  |  |  |  |  |  |
| Spariro test | **W** | **p-value** |  |  |  |  |
|  | 0.71661 | 2.2e-16 |  |  |  |  |
|  |  |  |  |  |  |  |
| 2 Way Anova | **Df** | **Sum Sq** | **Mean Sq** | **F value** | **P value** |  |
| species | 1 | 3.46 | 3.4634 | 4.5319 | **0.0338** | * |
| day | 17 | 19.31 | 1.1356 | 1.4859 | 0.0949 |  |
| Species*day | 17 | 20.19 | 1.1877 | 1.5541 | 0.0726 |  |
| Residual | 462 | 353.08 | 0.7642 |  |  |  |

**Statistics relative longest neurite (axon) length of bipolar iNs.**

| Days of differentiation | Species | N | Differences in % | Mean [µm] | SD [µm] | P value  (species) |
| --- | --- | --- | --- | --- | --- | --- |
| d7 | ape | 38 | +11% | 1192 | 529 | 0.2763 |
|  | human | 92 |  | 1323 | 705 |  |
| d14 | ape | 21 | -51% | 6859 | 11701 | **0.0426**  ***** |
|  | human | 33 |  | 3347 | 969 |  |
| d21 | ape | 22 | +38% | 8665 | 9518 | 0.3413 |
|  | human | 29 |  | 11958 | 7464 |  |
| d35 | ape | 9 | +32% | 18356 | 10778 | 0.2437 |
|  | human | 4 |  | 24274 | 14934 |  |
|  |  |  |  |  |  |  |
| Spariro test | **W** | **P value** |  |  |  |  |
|  | 0.7002 | 2.2e-16 |  |  |  |  |
|  |  |  |  |  |  |  |
| 2 Way Anova | **Df** | **Sum Sq** | **Mean Sq** | **F value** | **P value** |  |
| species | 1 | 0.061 | 0.06088 | 0.1177 | 0.7319 |  |
| day | 13 | 30.318 | 2.33219 | 4.5076 | **8.280e-07** | *** |
| Species*day | 13 | 28.536 | 2.19505 | 4.2423 | **2.531e-06** | *** |
| Residual | 220 | 113.826 | 0.51739 |  |  |  |

**Statistics relative number of sholl intersections of multipolar iNs.**

| Days of differentiation | Species | N | Differences in % | Mean | SD | P value  (species) |
| --- | --- | --- | --- | --- | --- | --- |
| d7 | ape | 45 | -18% | 5.7500 | 3.5937 | 0.1419 |
|  | human | 84 |  | 4.7349 | 2.0487 |  |
| d14 | ape | 78 | -17% | 7.5402 | 3.6593 | **0.0469**  ***** |
|  | human | 49 |  | 6.2444 | 2.6642 |  |
| d21 | ape | 93 | -1% | 8.3438 | 4.0570 | 0.4445 |
|  | human | 81 |  | 8.2577 | 3.9430 |  |
| d35 | ape | 32 | +4% | 11.5455 | 4.6976 | 0.5414 |
|  | human | 28 |  | 12.0000 | 6.0245 |  |
|  |  |  |  |  |  |  |
| Shapiro test | **W** | **P value** |  |  |  |  |
|  | 0.97969 | 8.769e-07 |  |  |  |  |
|  |  |  |  |  |  |  |
| 2 Way Anova | **Df** | **Sum Sq** | **Mean Sq** | **F value** | **P value** |  |
| species | 1 | 0.3936 | 0.39359 | 8.0897 | **0.0046** | ** |
| day | 3 | 7.1105 | 2.37016 | 48.7148 | **<2.2e-16** | *** |
| Species*day | 3 | 0.1441 | 0.04803 | 0.9872 | 0.3984 |  |
| Residual | 527 | 25.6405 | 0.04865 |  |  |  |

**Statistics relative number of sholl intersections of bipolar iNs.**

| Days of differentiation | Species | N | Differences in % | Mean | SD | P value  (species) |
| --- | --- | --- | --- | --- | --- | --- |
| d7 | ape | 38 | -14% | 4.9608 | 2.9797 | 0.1043 |
|  | human | 92 |  | 4.2456 | 2.1351 |  |
| d14 | ape | 21 | -26% | 6.5714 | 2.5593 | **0.0008**  ******* |
|  | human | 33 |  | 4.8679 | 3.4027 |  |
| d21 | ape | 22 | -10% | 8.3846 | 5.4851 | 0.4256 |
|  | human | 29 |  | 7.6176 | 4.1048 |  |
| d35 | ape | 9 | -13% | 12.4615 | 7.9332 | 0.4137 |
|  | human | 4 |  | 10.875 | 4.6117 |  |
|  |  |  |  |  |  |  |
| Spariro test | **W** | **P value** |  |  |  |  |
|  | 0.97347 | 1.011e-05 |  |  |  |  |
|  |  |  |  |  |  |  |
| 2 Way Anova | **Df** | **Sum Sq** | **Mean Sq** | **F value** | **P value** |  |
| species | 1 | 1.1079 | 1.10787 | 17.0543 | **4.644e-05** | *** |
| day | 3 | 4.4735 | 1.49118 | 22.9548 | **1.761e-13** | *** |
| Species*day | 3 | 3.0.2924 | 0.09746 | 1.5003 | 0.2144 |  |
| Residual | 319 | 20.7227 | 0.06496 |  |  |  |

**Table 7:** **Statistics total dendrite length of multipolar iNs.**

| Days of differentiation | Species | N | Differences in % | Mean [µm] | SD [µm] | P value  (species) |
| --- | --- | --- | --- | --- | --- | --- |
| d7 | ape | 45 | -20% | 776 | 101 | 0.1226 |
|  | human | 84 |  | 618 | 84.4 |  |
| d14 | ape | 78 | -72% | 2740 | 666 | **0.0009 ***** |
|  | human | 49 |  | 770 | 136 |  |
| d21 | ape | 93 | -12% | 6843 | 1406 | 0.7268 |
|  | human | 81 |  | 6001 | 905 |  |
| d35 | ape | 32 | -41% | 13356 | 6113 | 0.3336 |
|  | human | 28 |  | 7926 | 3328 |  |
|  |  |  |  |  |  |  |
| Shapiro test | **W** | **P value** |  |  |  |  |
|  | 0.99504 | 0.1111 |  |  |  |  |
|  |  |  |  |  |  |  |
| 2 Way Anova | **Df** | **Sum Sq** | **Mean Sq** | **F value** | **P value** |  |
| species | 1 | 4.331 | 4.3312 | 10.3340 | **0.0014** | ** |
| day | 3 | 59.997 | 19.9989 | 47.7157 | **<2.2e-16** | *** |
| Species*day | 3 | 3.074 | 1.0248 | 2.4451 | 0.0633 |  |
| Residual | 490 | 205.372 | 0.4191 |  |  |  |

**Statistics total dendrite length of bipolar iNs.**

| Days of differentiation | Species | N | Differences in % | Mean | SD | P value  (species) |
| --- | --- | --- | --- | --- | --- | --- |
| d7 | ape | 38 | -13% | 505 | 71.8 | 0.2874 |
|  | human | 92 |  | 440 | 49.2 |  |
| d14 | ape | 21 | -89% | 2918 | 1404 | **0.0447**  ***** |
|  | human | 33 |  | 332 | 77.2 |  |
| d21 | ape | 22 | -11% | 3630 | 1214 | 0.8698 |
|  | human | 29 |  | 3234 | 896 |  |
| d35 | ape | 9 | -43% | 3175 | 888 | 0.7489 |
|  | human | 4 |  | 1821 | 129 |  |
|  |  |  |  |  |  |  |
| Spariro test | **W** | **P value** |  |  |  |  |
|  | 0.99165 | 0.1712 |  |  |  |  |
|  |  |  |  |  |  |  |
| 2 Way Anova | **Df** | **Sum Sq** | **Mean Sq** | **F value** | **P value** |  |
| species | 1 | 3.403 | 3.4033 | 7.4732 | **0.006729** | ** |
| day | 3 | 15.794 | 5.2648 | 11.5608 | **4.184e-07** | *** |
| Species*day | 3 | 3.1.552+08 | 0.5172 | 1.1358 | 0.335257 |  |
| Residual | 240 | 109.296 | 0.04554 |  |  |  |
