# Supplementary material for: Comparison of induced neurons reveals slower structural and functional maturation in humans than in apes
Source: eLife. 2021 Jan 20;10:e59323. doi: 10.7554/eLife.59323 (PMC7870144; doi:10.7554/eLife.59323)
Supplement: Transparent reporting form [file elife-59323-transrepform.docx]

***eLife’s* transparent reporting form**

We encourage authors to provide detailed information *within their submission* to facilitate the interpretation and replication of experiments. Authors can upload supporting documentation to indicate the use of appropriate reporting guidelines for health-related research (see [EQUATOR Network](http://www.equator-network.org/%20)), life science research (see the [BioSharing Information Resource](https://biosharing.org/)), or the [ARRIVE guidelines](http://www.plosbiology.org/article/info:doi/10.1371/journal.pbio.1000412) for reporting work involving animal research. Where applicable, authors should refer to any relevant reporting standards documents in this form.

If you have any questions, please consult our Journal Policies and/or contact us: [editorial@elifesciences.org](mailto:editorial@elifesciences.org).

**Sample-size estimation**

- You should state whether an appropriate sample size was computed when the study was being designed
- You should state the statistical method of sample size computation and any required assumptions
- If no explicit power analysis was used, you should describe how you decided what sample (replicate) size (number) to use

Please outline where this information can be found within the submission (e.g., sections or figure legends), or explain why this information doesn’t apply to your submission:

We have outlined the information on sample size and statistical methods used in the Material and Methods Section. Specific detailed information on sample size and statistical methods used can also be found on Supplementary table 1, 3 and 4 and on the Figure legends (Figure 2, 4, 5 and Figure 5_Figure Supplement 2).

**Replicates**

- You should report how often each experiment was performed
- You should include a definition of biological versus technical replication
- The data obtained should be provided and sufficient information should be provided to indicate the number of independent biological and/or technical replicates
- If you encountered any outliers, you should describe how these were handled
- Criteria for exclusion/inclusion of data should be clearly stated
- High-throughput sequence data should be uploaded before submission, with a private link for reviewers provided (these are available from both GEO and ArrayExpress)

Please outline where this information can be found within the submission (e.g., sections or figure legends), or explain why this information doesn’t apply to your submission:

We have outlined the information on replicates and inclusion/exclusion of data in the Material and Methods Section. We refer to technical replicates as performed experiments. Detailed information about the number of samples can be found in Supplementary File 3 (for electrophysiological data) and Supplementary File 4 (for morphological data). For the electrophysiological data 1-8 technical replicates have been generated per cell line (Replicates: N_hiPS-409-B2_=8, N_SC102A1_=3, N_HmRNA_=2, N_H9_=2, N_SandraA_=8, N_JoC_=2, N_ciPS01_=2, N_BmRNA_=1). For each experiment several time points and cells have been analyzed. For the morphological analysis 4-9 technical replicates have been used to generate the raw data and for the normalization of the axon length (Replicates: N_d7_=7, N_d14_=5, N_d21_=9, N_d21_=4). For each replicate several time points, cell lines and individual cells have been analyzed. Outliers were included into the analysis. High-throughput sequence data source for single cells are available at ArrayExpress: E-MTAB-9233 and on Mendeley Data with doi: 10.17632/y3s4hnyvg6 (Technical Replicates: N_iPS_ = 1, N_d5_ = 2, N_d14_ = 1 , N_d28/d29_ = 3, and N_d35_ = 2 for hiPS-409-B2 cells; N_d35_ = 1 for H9 cells; N_d35_ = 1 for SC102A1 cells; N_ips_ = 1, N_d5_ = 2, N_d14_ = 2, N_d28/d29_ = 3, and N_d35_ = 5 for SandraA cells.)

All batches and respective number of cells analyzed is listed in a separate table (Supplementary File 5).

**Statistical reporting**

- Statistical analysis methods should be described and justified
- Raw data should be presented in figures whenever informative to do so (typically when N per group is less than 10)
- For each experiment, you should identify the statistical tests used, exact values of N, definitions of center, methods of multiple test correction, and dispersion and precision measures (e.g., mean, median, SD, SEM, confidence intervals; and, for the major substantive results, a measure of effect size (e.g., Pearson's r, Cohen's d)
- Report exact p-values wherever possible alongside the summary statistics and 95% confidence intervals. These should be reported for all key questions and not only when the p-value is less than 0.05.

Please outline where this information can be found within the submission (e.g., sections or figure legends), or explain why this information doesn’t apply to your submission:

We have outlined the information on statistical methods used in the Material and Methods Section. Specific detailed information on statistical methods used, can also be found on Supplementary File 3 and 4 and on the Figure legends (Figure 4, 5 and Figure 5_Figure Supplement 2).

(For large datasets, or papers with a very large number of statistical tests, you may upload a single table file with tests, Ns, etc., with reference to sections in the manuscript.)

**Group allocation**

- Indicate how samples were allocated into experimental groups (in the case of clinical studies, please specify allocation to treatment method); if randomization was used, please also state if restricted randomization was applied
- Indicate if masking was used during group allocation, data collection and/or data analysis

Please outline where this information can be found within the submission (e.g., sections or figure legends), or explain why this information doesn’t apply to your submission:

Induced neurons (samples) derived from human, chimpanzee and bonobo stem cells, were allocated to different groups determined by their taxonomic category genus (*Homo* or *Pan*) and the day after induction (d7; d14; d21; d28; d35).

**Additional data files (“source data”)**

- We encourage you to upload relevant additional data files, such as numerical data that are represented as a graph in a figure, or as a summary table
- Where provided, these should be in the most useful format, and they can be uploaded as “Source data” files linked to a main figure or table
- Include model definition files including the full list of parameters used
- Include code used for data analysis (e.g., R, MatLab)
- Avoid stating that data files are “available upon request”

Please indicate the figures or tables for which source data files have been provided:

We have provided the High-throughput RNA sequence data source for single cells: ArrayExpress: E-MTAB-9233 and Mendeley Data with doi: 10.17632/y3s4hnyvg6

To make our scRNAseq data accessible to the neuroscience community, we provide a ShinyApp-based web browser for data exploration, called iNeuronExplorer.

*https://bioinf.eva.mpg.de/shiny/iNeuronExplorer/*

We provided the script for analysis and raw data for the morphological analysis under the URL: https://github.com/BenjaminPeter/schornig_ineuron.
